# Electron probe X-ray microanalysis of boar and inobuta testes after the Fukushima accident

**DOI:** 10.1093/jrr/rrv070

**Published:** 2016-01-29

**Authors:** Hideaki Yamashiro, Yasuyuki Abe, Gohei Hayashi, Yusuke Urushihara, Yoshikazu Kuwahara, Masatoshi Suzuki, Jin Kobayashi, Yasuyuki Kino, Tomokazu Fukuda, Bin Tong, Sachio Takino, Yukou Sugano, Satoshi Sugimura, Takahisa Yamada, Emiko Isogai, Manabu Fukumoto

**Affiliations:** 1 Faculty of Agriculture, Niigata University, Niigata, Japan; 2 Research Centre for Protozoan Diseases, Obihiro University of Agriculture and Veterinary Medicine, Obihiro, Japan; 3 Institute of Development, Aging and Cancer, Tohoku University, Sendai, Japan; 4 School of Food, Agricultural and Environmental Sciences, Miyagi University, Sendai, Japan; 5 Graduate School of Science, Tohoku University, Sendai, Japan; 6 Graduate School of Agricultural Sciences, Tohoku University, Sendai, Japan; 7 Institute of Agriculture, Department of Biological Production, Tokyo University of Agriculture and Technology, Tokyo, Japan

**Keywords:** boar, EPMA, Fukushima Daiichi Nuclear Power Plant, inobuta, radioactive caesium, testis

## Abstract

We aimed to investigate the effect of chronic radiation exposure associated with the Fukushima Daiichi Nuclear Power Plant (FNPP) accident on the testes of boar and inobuta (a hybrid of *Sus scrofa* and *Sus scrofa domestica*). This study examined the contamination levels of radioactive caesium (Cs), especially ^134^Cs and ^137^Cs, in the testis of both boar and inobuta during 2012, after the Fukushima accident. Morphological analysis and electron-probe X-ray microanalysis (EPMA) were also undertaken on the testes. The ^134^Cs and ^137^Cs levels were 6430 ± 23 and 6820 ± 32 Bq/kg in the boar testes, and 755 ± 13 and 747 ± 17 Bq/kg in the inobuta testes, respectively. The internal and external exposure of total ^134^Cs and ^137^Cs in the boar testes were 47.1 mGy and 176.2 mGy, respectively, whereas in the inobuta testes, these levels were 6.09 mGy and 59.8 mGy, respectively. Defective spermatogenesis was not detected by the histochemical analysis of radiation-exposed testes for either animal. In neither animal were Cs molecules detected, using EPMA. In conclusion, we showed that adverse radiation-induced effects were not detected in the examined boar and inobuta testes following the chronic radiation exposure associated with the FNPP accident.

## INTRODUCTION

The Fukushima Daiichi Nuclear Power Plant (FNPP) accident, which occurred after the Great East Japan Earthquake on 11 March 2011, led to the discharge of radioactive substances. The presence of ^134^Caesium (Cs) and ^137^Cs, which emit γ- and β-rays, was of primary concern, because they were released in large quantities and have a long half-life [1]. Therefore, significant questions about the effects of long-term exposure to radioactive Cs on human health have been raised.

We recently reported radionuclide deposition in the organs of abandoned cattle after the FNPP accident. The deposition of an individual radionuclide occurred in an organ-specific manner, with radioactive Cs being detected in all examined organs [2]. Electron-probe X-ray microanalysis (EPMA) is a powerful tool used to detect trace amounts of chemical elements in single cells and tissues [3]. This method measures the characteristic X-ray spectra of specific elements in samples using an accelerated electron beam. We previously investigated the effects of chronic radiation exposure to ^134^Cs and ^137^Cs of the testis from euthanized bulls in the evacuation zone [4]. Radioactivity concentrations of ^134^Cs and ^137^Cs in bull organs were detected by γ-ray spectrometry. However, we failed to detect Cs molecules by EPMA because the contamination level was lower than the detection limit in the testes of the examined bulls.

Here, we evaluated the contamination levels of radioactive caesium levels, especially ^134^Cs and ^137^Cs levels, in the testes of boar and inobuta (a hybrid of *Sus scrofa* and *Sus scrofa domestica*), both of which are economically important animals for the livestock industry. We collected the organs and testes from euthanized male boar and inobuta on 18 January and 28 February 2012. Morphological and EPMA analyses were used in the evaluation. Information about effects of radiation on farm animals in the evacuation zone after the FNPP accident helps us to understand the health risks of livestock to humans, as radiation may pass from one to the other during consumption. No previous studies have investigated the effects of chronic radiation on the reproductive organs of boar and inobuta in the period after the Fukushima accident.

## MATERIALS AND METHODS

### Collection of samples

We obtained the testes from euthanized boar and inobuta in collaboration with the combined unit of veterinary doctors from the Livestock Hygiene Service Centre of Fukushima Prefecture, Japan. The boar samples were collected on 18 January 2012 from Tomioka Town, located 5 km southwest of FNPP, where the dose rate in the air was 10 μSv/h. The inobuta samples were collected on 28 February 2012 from another location in Tomioka Town, located 8 km southwest of FNPP, where the dose rate in air was 15 μSv/h. Estimates of the amounts of ^134^Cs and ^137^Cs deposited on the ground were calculated from the air dose rates (Table 1). The Ethics Committee of Animal Experiments, Tohoku University, Japan, approved this study.

**Table 1.** Estimates of the amount of ^134^Cs and ^137^Cs-deposited on the ground were calculated from the air dose rates (kBq/m^2^)

Animal	Date	^134^Cs	^137^Cs
Boar	18 January 2012	3218	3250
Inobuta	28 February 2012	976	986

### Measurements of radioactivity

The radioactivity of the samples was determined by gamma-ray spectrometry using three High-purity Germanium (HPGe) detectors (Ortec Co., Atlanta, USA). A nuclide was identified when characteristic photopeaks greater than 3σ were observed above the baseline in the spectrum. Based on the count rate from each sample, we calculated the concentration of these radionuclides (Bq/kg sample). Efficiency curves for the detectors were obtained by using standard gel sources containing known amounts of ^152^Eu and ^137^ Cs. These measurements were carried out from January to March 2012. All measurements were decay-corrected to the day of the radioactivity release, i.e. 11 March 2011 [5].

### Calculation of internal and external dose rates

The dose rates of internal and external exposure to ^134^Cs and ^137^Cs were calculated according to the method of dosimetry assumption described in ICRP Publication 108 [6].

We made several assumptions in the estimation of internal dose rates. The average radioactivity concentration of the total body was calculated from the radioactivity concentrations of the examined organs. We used body length and the depth and width of the chest to calculate the conversion coefficient for the dose rate.

The external dose rate was calculated from ^134^Cs and ^137^Cs concentrations in the soil, assuming a certain distance between the soil surface and the examined body parts. The external exposure of ^134^Cs and ^137^Cs over specific durations was calculated [7].

### Morphological assessment of testes cells

The testes were fixed in Bouin's solution, embedded in paraffin, and stained using haematoxylin and eosin (HE), according to the standard protocols described by Akiyama *et al.* [8]. Subsequently, the testes were briefly dehydrated in a series of different concentrations of alcohol, made transparent using toluene, embedded in paraffin and cut into 5-μm-thick sections before staining.

### Electron probe X-ray microanalysis

Chemical trace analyses of Cs, C, K and Mo in the testes were performed using a JEOL JXA-8230 superprobe electron probe microanalyzer (JEOL, Tokyo, Japan) equipped for X-ray spectrometry and specifically adapted for the examination of ultrathin sections [4]. The specimens were mounted on a silicon wafer, and an electron beam of 0.3 μm diameter was used for focusing. For the analysis, the voltage of the electron microscope was set to 15 kV and the electron beam rate was set to 1 μA. The sections were viewed as secondary electron images, and chemical elemental mapping was performed.

## RESULTS AND DISCUSSION

The testis is one of the most radiosensitive tissues, with low doses of radiation causing significant impairment of function. Damage may be caused even during direct irradiation of the testis [9]. Immature cells are more radiosensitive, even to doses as low as 0.1 Gy, causing morphological and quantitative changes to the spermatogonia in the testis. Doses of 2–3 Gy cause overt damage to spermatocytes, reducing spermatid numbers. At doses of 4–6 Gy, spermatozoa numbers significantly decrease, implying damage to the spermatids.

The ^134^Cs and ^137^Cs radioactivity levels in boar and inobuta testes were measured by γ-ray spectrometry using three HPGe detectors (Table 2) and were found to be very similar in both animals. The ^134^Cs and ^137^Cs levels in boar testes were 6430 ± 23 and 6820 ± 32 Bq/kg, respectively, while those of the tensor fasciae late muscle were 16 600 ± 110 and 17 700 ± 148 Bq/kg, respectively. In inobuta, the ^134^Cs and ^137^Cs levels were 753 ± 13 and 747 ± 17 Bq/kg, respectively, in the testes and 1070 ± 20 and 1010 ± 25 Bq/kg in the tensor fasciae late muscle.

**Table 2.** Radioactivity concentration of ^134^Cs and ^137^Cs in the testis and tensor fasciae late muscle (Bq/kg)

Animal	Testis		Tensor fasciae late muscle	
	^134^Cs	^137^Cs	^134^Cs	^137^Cs
Boar	6430 ± 23	6820 ± 32	16 600 ± 110	17 700 ± 148
Inobuta	753 ± 13	747 ± 17	1070 ± 20	1010 ± 25

**Fig. 1.**
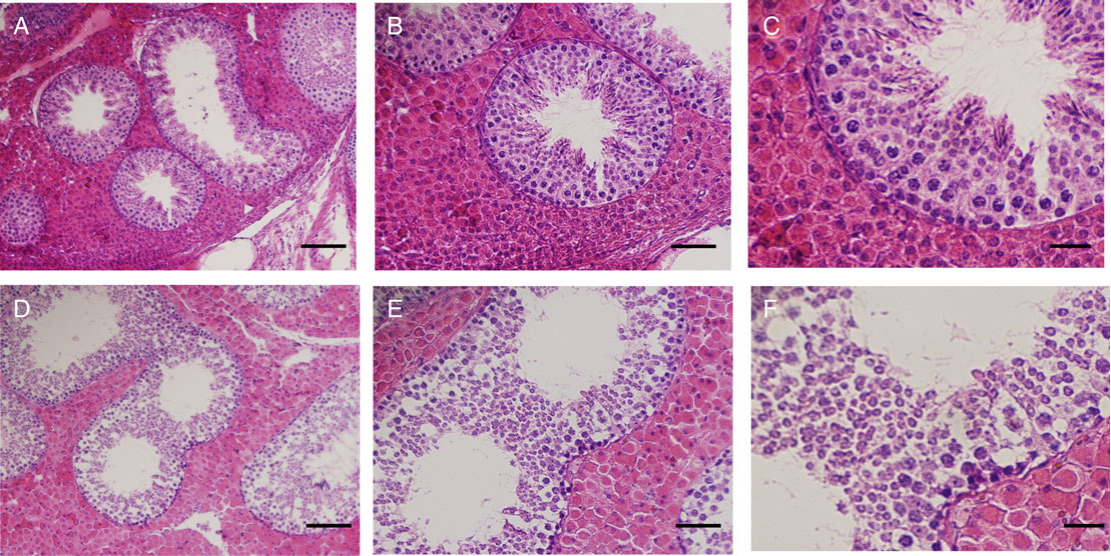
Histological sections of the seminiferous tubules of radiation-exposed testis for boar (**A–C**) and inobuta (**D–F**). Scale bar: 100 μm in A and D; 50 μm in B and E; 20 μm in C and F.

Table 3 shows that the internal and external exposure of total ^134^Cs and ^137^Cs in the boar testis was 47.1 and 176.2 mGy, respectively. In comparison, these levels were 6.09 and 59.8 mGy, respectively, in the inobuta testis. Despite being exposed to radiation for 10–11 months, the cellular associations (i.e. multiple seminiferous tubules lined with spermatogonia, spermatocytes, spermatids and sperm) in the boar and inobuta testes indicated normal spermatogenesis (Fig. 1A–F). In addition, the sperm cell numbers in the testes of radiation-exposed animals showed normal spermatogenesis in the seminiferous tubules.

**Table 3.** Internal and external exposure of ^134^Cs and ^137^Cs in the testis and tensor fasciae late muscle (mGy)

Animal	Internal				External	
	Testis		Tensor fasciae late muscle			
	^134^Cs	^137^Cs	^134^Cs	^137^Cs	^134^Cs	^137^Cs
Boar	29.8	17.3	77.0	44.9	124.1	52.1
Inobuta	3.95	2.14	5.6	2.9	41.9	17.9

**Fig. 2.**
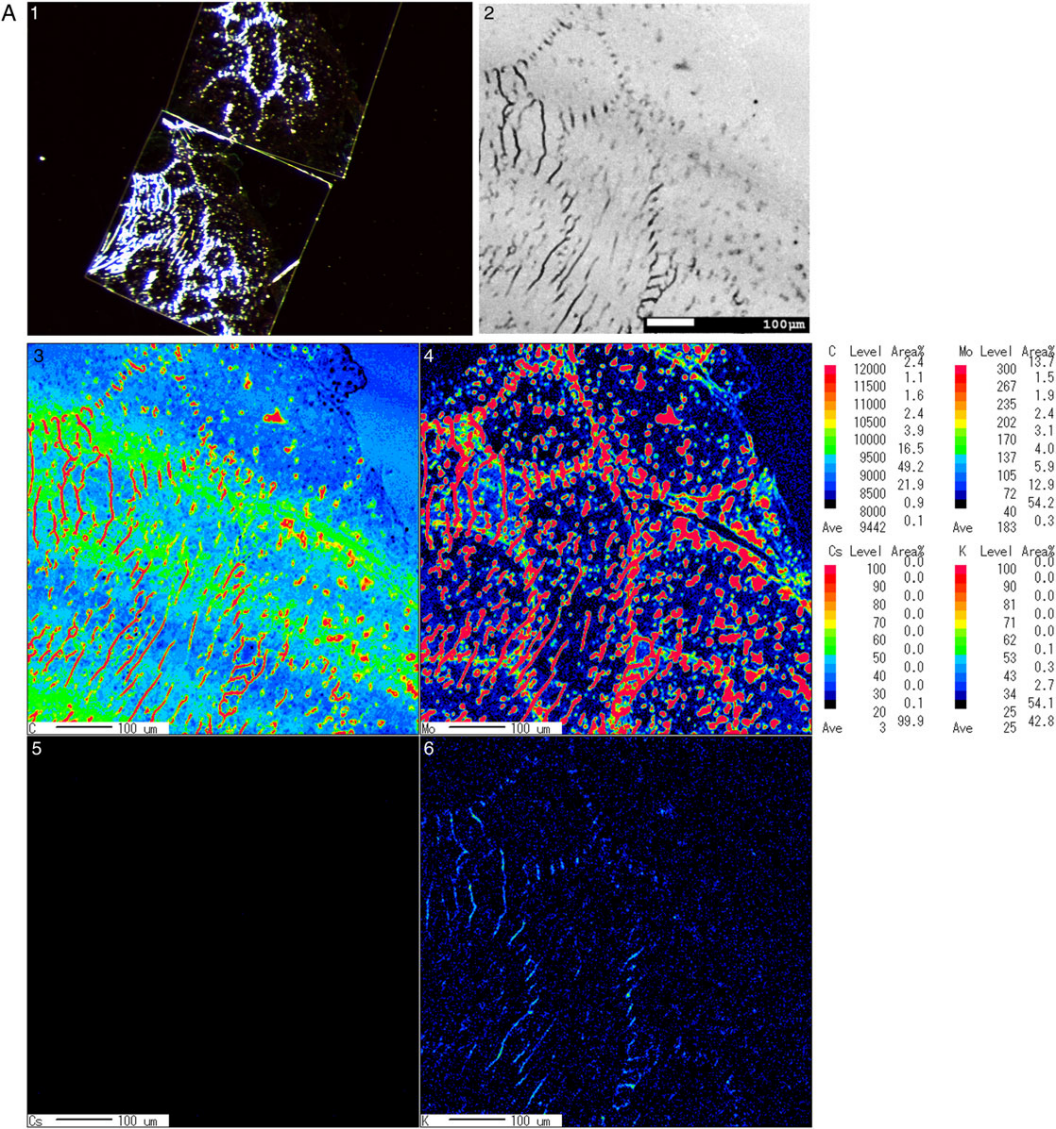

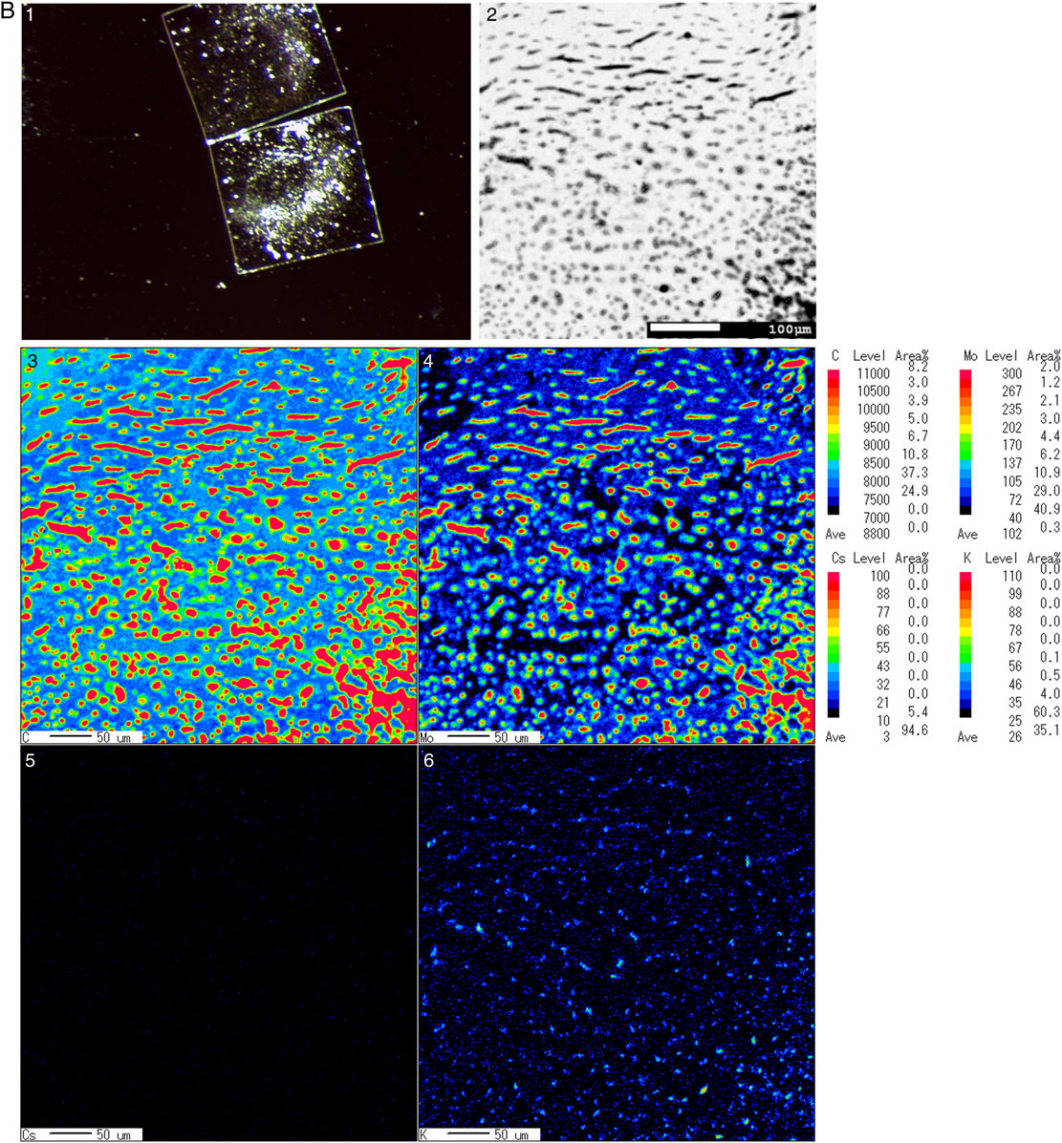
EPMA analysis of boar and inobuta testis. **A**. Boar, **B**. Inobuta. 1. Stereo-microscopy images of testis. 2. Composite backscattered electron microscopy images. 3. Secondary electron colour map image of C (carbon) regions. 4. Corresponding distribution of Mo (molybdenum) obtained from the same section. 5. X-ray colour-coded phase map of Cs (caesium). 6. Corresponding X-ray profiles for K (potassium).

EPMA is a powerful tool for detecting trace chemical elements in single cells and tissues via measurement of the characteristic X-ray spectra of specific elements in samples by using an accelerated electron beam. Figure 2A–B: slides 3, 4, 5 and 6 present phase maps indicating microconstituent concentrations; namely, C, Mo, K and Cs, respectively. Colour imaging rapidly and effectively facilitates the overall analysis of the composite structure; specifically, decreasing levels of metal distribution are indicated from red to black. In the boar samples, an intermediate imaging result (light blue and green) was obtained for C in the seminiferous tubules (Fig. 2A: slide 3). However, Mo was detected around the seminiferous tubules, and K levels were low (Fig. 2A: slides 4 and 6). In the inobuta testis, high to intermediate C imaging results were obtained (red and light green) for the seminiferous tubules, whereas both Mo and K levels were low (dark blue; Fig. 2B: slides 3, 4 and 6). Cs was not detected in the testes of either boar or inobuta (Fig. 2A–B: slide 5).

A recent study on mice showed that low-dose-rate radiation exposure (3.5 mGy/h) caused no adverse effects at dose levels of ≤2 Gy; however, the testis weight and sperm count and motility decreased at a dose of 2 Gy [10]. To our knowledge, previous studies on boar and inobuta have not shown that low-dose-rate radiation exposure causes damage to the testes; consequently, no definitive proof is available.

Our results showed that the radioactivity levels of ^137^Cs in the boar testis and tensor fasciae late muscle were approximately 9- and 17.5-fold higher than those detected in inobuta. However, HE staining showed no histological difference between the testis of the boar and inobuta, with normal spermatogenesis being observed in the seminiferous tubules. This result indicates that chronic exposure to radiation at this level did not affect male germ cell morphology in either boar or inobuta.

In conclusion, we showed that adverse radiation-induced effects were not detected in the examined boar and inobuta testes following chronic radiation exposure associated with the FNPP accident.

## FUNDING

This work was partly supported by a grant awarded to MF by the Japan Society for the Promotion of Science, Japan. The research was also supported by a grant awarded to EI by the Research and Development Projects for Application in Promoting New Policy of Agriculture, Forestry and Fisheries, Ministry of Agriculture, Forestry and Fisheries (MAFF), Japan, and the Programme for Promotion of Basic and Applied Researches for Innovations in Bio-oriented Industry (BRAIN), Japan. HY was supported in this work through the Specific Research Grant 2014 for the East Japan Great Earthquake Revival by The New Technology Development Foundation. Funding to pay the Open Access publication charges for this special issue was provided by the Grant-in-Aid from the Japan Society for the Promotion of Science (JSPS) [KAKENHI Grant No. 26253022].
